# Optimal implementation of genomic selection in clone breeding programs exemplified in potato: II. Effect of selection strategy and cross‐selection method on long‐term genetic gain

**DOI:** 10.1002/tpg2.70000

**Published:** 2025-02-18

**Authors:** Po‐Ya Wu, Benjamin Stich, Stefanie Hartje, Katja Muders, Vanessa Prigge, Delphine Van Inghelandt

**Affiliations:** ^1^ Institute of Quantitative Genetics and Genomics of Plants Heinrich Heine University Düsseldorf Germany; ^2^ Institute for Breeding Research on Agricultural Crops Federal Research Centre for Cultivated Plants Sanitz Germany; ^3^ Cluster of Excellence on Plant Sciences (CEPLAS) Heinrich Heine University Düsseldorf Germany; ^4^ Max Planck Institute for Plant Breeding Research Köln Germany; ^5^ Böhm‐Nordkartoffel Agrarproduktion GmbH & Co. OHG Lüneburg Germany; ^6^ NORIKA GmbH Sanitz Germany; ^7^ SaKa Pflanzenzucht GmbH & Co. KG Windeby Germany; ^8^ Department of Genebank Leibniz Institute of Plant Genetics and Crop Plant Research Sanitz Germany

## Abstract

Different cross‐selection (CS) methods incorporating genomic selection (GS) have been used in diploid species to improve long‐term genetic gain and preserve diversity. However, their application to heterozygous and autotetraploid crops such as potato (*Solanum tuberosum* L.) is lacking so far. The objectives of our study were to (i) assess the effects of different CS methods and the incorporation of GS and genetic variability monitoring on both short‐ and long‐term genetic gains compared to strategies using phenotypic selection (PS); (ii) evaluate the changes in genetic variability and the efficiency of converting diversity into genetic gain across different CS methods; and (iii) investigate the interaction effects between different genetic architectures and CS methods on long‐term genetic gain. In our simulation results, implementing GS with optimal selected proportions had increased short‐ and long‐term genetic gain compared to any PS strategy. The CS method considering additive and dominance effects to predict progeny mean based on simulated progenies (MEGV‐O) achieved the highest long‐term genetic gain among the assessed mean‐based CS methods. Compared to MEGV‐O and usefulness criteria (UC), the linear combination of UC and genome‐wide diversity (called EUCD) maintained the same level of genetic gain but resulted in higher diversity and a lower number of fixed QTLs. Moreover, EUCD had a relatively high degree of efficiency in converting diversity into genetic gain. However, choosing the most appropriate weight to account for diversity in EUCD depends on the genetic architecture of the target trait and the breeder's objectives. Our results provide breeders with concrete methods to improve their potato breeding programs.

## Core Ideas

- Optimized genomic selection scheme reached higher short‐ and long‐term genetic gain than phenotypic selection.
- Cross‐selection methods based on progeny mean with dominance effects achieved the highest long‐term genetic gain.
- Combining the usefulness criterion (UC) and extended usefulness criterion incorporating genomic diversity index (EUCD) could reach similar high long‐term genetic gain.
- EUCD could simultaneously maintain a higher genetic diversity than progeny mean‐based and UC methods.

**Abbreviations**
A: A clone stage
B: B clone stage
C: C clone stage
D: D clone stage
CS method: cross‐selection method
$C_{0}$
: burn‐in cycle
$C_{1}$
: cycle 1
$C_{t}$
: cycle *t*
EBV: estimated breeding values
EGV: estimated genetic values
EUC: extended usefulness criterion, incorporating different weight ($w_{1}$) on the progeny variance
EUCD: extended usefulness criterion incorporating genomic diversity index
GS: genomic selection
H
: square root of the heritability
He: expected heterozygosity
i
: selection intensity
MEBV‐O: mean estimated breeding values among simulated offspring
MEBV‐P: mean estimated breeding values of the two parents
MEGV‐O: mean estimated genetic values among simulated offspring
MEGV‐P: mean estimated genetic values of the two parents
MPV: mean phenotypic values of the two parents
OCS: optimal cross‐selection
P: phenotypic values
$p_{i}$
: selection proportion at the $i^{\mathrm{th}}$ stage
PA: prediction accuracy of the GS model
PS: phenotypic selection
QTL: quantitative trait locus
SL: seedling stage
SH: single hill stage
$T_{a}$
: auxiliary trait
$T_{t}$
: target trait
TBV: true breeding values
TGV: true genetic values
UC: usefulness criterion
μ
: progeny mean
X: cross stage
$\sigma_{G}$
: square root of the progeny variance

## 1 INTRODUCTION

Potato (*Solanum tuberosum* L.) is one of the most important non‐cereal crops for human consumption in the world (http://www.fao.org/faostat/en/). With the global population growing, producing sufficient food is becoming a big challenge for agriculture (Fróna et al., 2019). In addition, global crop production is expected to be negatively impacted by climate change due to an increase in extreme temperatures and an alternation of rainfall patterns (Abberton et al., 2016). Thus, developing methods and approaches which increase the efficiency and effectiveness of creating improved and adapted potato varieties is one of the important tasks of plant geneticists.

One necessary step for developing varieties is the generation of new genetic variability. This can be achieved by (1) introducing new alleles, for instance using genetic resource collections (Sanchez et al., 2023) and (2) creating new allelic combinations. The latter happens during meiotic recombinations that occur after crossing parental genotypes to create segregating populations. Subsequently, superior clones are identified in multi‐year testing as variety candidates and new cross combinations are determined to start the next breeding cycle. In a typical clonal breeding program, these steps have hitherto relied mostly on phenotypic selection, which takes several years. This is especially true for potato crops, because most target traits can only be assessed in the later stages due to the crop's low multiplication coefficient (Grüneberg et al., 2009), which in turn hampers the increase of genetic gain.

Recently, genomic selection (GS) has been shown to enhance genetic gain in both livestock and crop breeding (Alemu et al., 2024). In potato, Wu et al. (2023) have shown via computer simulations that implementing GS into one breeding cycle can improve short‐term genetic gain of the target trait compared to using phenotypic selection (PS). While incorporating GS into breeding programs has been shown to increase long‐term genetic gain in diploid crops compared to PS (Gaynor et al., 2017; Gorjanc et al., 2018; Lubanga et al., 2022; Muleta et al., 2019; Sanchez et al., 2023; Werner et al., 2023), the effects of implementing GS on long‐term genetic gain in autotetraploid and heterozygous crops are still unclear. Furthermore, due to the complicated quantitative genetics and the importance of dominance effects in the latter, different trends in implementation of GS can be expected compared to diploid crops, which need to be assessed.

The genetic value of new crosses is commonly predicted by the mid‐parental performance based on the phenotypic records of candidate parents (Brown & Caligari, 1989). With GS, this genetic value can be estimated from a trained GS model as estimated genetic values (EGV). This has been shown to improve genetic gain in maize compared to phenotypic assessment (Allier et al., 2019; Sanchez et al., 2023). However, as GS is also a truncation selection, it is accompanied by an acceleration of the fixation of favorable alleles. This is because the candidate parents that are intermated in order to create the next generation have similar genetic backgrounds, which hinders the generation of new allelic recombinations and limits the long‐term improvement of genetic gain (cf. Jannink, 2010). Therefore, preserving diversity in the breeding populations when implementing GS to select new crosses is an option for improving long‐term genetic gain.

Several studies have proposed different approaches to balance genetic gain and diversity while determining desirable new crosses. Daetwyler et al. (2015) proposed an optimal haploid value to predict the best homozygous line that can be generated from a cross. They showed that this approach can improve genetic gain and preserve genetic diversity better than truncation GS. However, the progenies of a cross in potato are highly heterozygous, meaning that the optimal haploid value is not effective in predicting their phenotypes. Schnell and Utz (1975) proposed the usefulness criterion (UC) to predict the performance of a cross. The UC considers the expected progeny mean (μ) and the expected response to selection ($\mathrm{iH}\sigma_{G}$) in the first generation progenies: $\mathrm{UC}=\mu +\mathrm{iH}\sigma_{G}$, where $\sigma_{G}$ is the square root of the progeny variance, i is the selection intensity, and H is the square root of the heritability. The UC approach has been shown to increase genetic gain compared to mid‐parental values in simulation studies on maize (Allier et al., 2019; Lehermeier et al., 2017; Sanchez et al., 2023). Furthermore, Zhong and Jannink (2007) made a modification of the UC, called superior progeny value: $S=\mu +i\sigma_{G}$. This focuses on progeny mean and variance but ignores heritability. However, depending on the traits, both UC and S can be close to the progeny mean as the variation in progeny mean is much higher than the variation in progeny standard deviation (Lado et al., 2017; Zhong & Jannink, 2007). This aspect limits the advantages of cross‐selection (CS) methods like UC and S. Therefore, investigating different weights between progeny mean and progeny variance could affect the efficiency of such CS methods on long‐term genetic gain. This, however, has not yet been studied.

The progeny mean of a biparental cross can be predicted by mid‐parental performance based on either phenotypic records or EGV from a trained GS model. This can be assessed for inbred populations derived from inbred parents or for hybrids and outbreds in the absence of dominance effects. For diploid species, the progeny mean can also be estimated in the presence of dominance effects (Falconer & Mackay, 1996; Wolfe et al., 2021; Werner et al., 2023). However, no formula is available to estimate the progeny mean for autotetraploid species. Furthermore, it is not easy to obtain a reliable prediction of progeny variance (Mohammadi et al., 2015). High density genome‐wide markers and GS models enable marker effects to be well estimated (Meuwissen et al., 2001). Recently, several formulae considering linkage disequilibrium and recombination rate in parental lines have been derived in order to predict the progeny variance (Allier et al., 2019; Bonk et al., 2016; Lehermeier et al., 2017; Osthushenrich et al., 2017; Wolfe et al., 2021). However, these formulae assume a diploid inheritance and thus cannot be applied to tetraploid potato.

**Core Ideas**

- Optimized genomic selection scheme reached higher short‐ and long‐term genetic gain than phenotypic selection.
- Cross‐selection methods based on progeny mean with dominance effects achieved the highest long‐term genetic gain.
- Combining the usefulness criterion (UC) and extended usefulness criterion incorporating genomic diversity index (EUCD) could reach similar high long‐term genetic gain.
- EUCD could simultaneously maintain a higher genetic diversity than progeny mean‐based and UC methods.

The simulation of virtual progenies of a cross using a genetic map and phased parental haplotype information is an alternative approach to address the lack of a formula considering autotetraploid inheritance (Bernardo, 2014; Mohammadi et al., 2015). Software for this purpose are available (e.g. AlphaSimR; Gaynor et al., 2021) and can be used for simulation in autotetraploid species. The use of average and variance of EGV among in silico progenies to predict progeny mean and variance could lead to more precise estimates in comparison to mid‐parental values. This approach would provide a solution to predict progeny variance for autotetraploid species with heterozygous parents. This aspect, however, has not previously been examined.

An alternative to UC and the derived methods is optimal cross‐selection (OCS) (Gorjanc et al., 2018). The basic idea of OCS is to select a group of biparental crosses that maximize the expected progeny mean under a certain constraint of genetic diversity or co‐ancestry on the selected population of individuals who serve as parents. Through optimization algorithms (e.g. Kinghorn, 2011), this approach has proven to increase long‐term genetic gain in a simulated maize breeding program with a minor penalty on short‐term genetic gain compared to using solely UC (Allier et al., 2019; Sanchez et al., 2023). However, it is substantially more time consuming to find the optimal parents and crosses compared to the abovementioned CS methods based on ranking the performance among all possible crosses, especially when many markers and candidates are used in autotetraploid breeding programs. This limits its utility, especially for potato breeding.

An alternative option to OCS for quantifying diversity can be based on the genome‐wide variation of a cross itself rather than the variation in the whole population of parents for crosses. This could be measured by the expected heterozygosity (He). Accounting for this element during the selection of new crosses may contribute to long‐term genetic gain and simultaneously preserve diversity while being computationally easy to realize. However, to the best of our knowledge, few studies have investigated the performance of such a criterion including the genome‐wide diversity of a cross to determine new desirable crosses.

The objectives of this study were to (i) assess the effects of different CS methods and the incorporation of GS and genetic variability monitoring on both short‐ and long‐term genetic gains compared to strategies using PS; (ii) evaluate the changes in genetic variability as well as the efficiency of converting diversity into genetic gain across different CS methods; and (iii) investigate the interaction effects between different genetic architectures and CS methods on long‐term genetic gain in polyploid clone breeding programs.

## 2 MATERIALS AND METHODS

### 2.1 Empirical genomic dataset for potato

For this simulation study, a set of 80 tetraploid potato clones, genotyped for 19,649,193 phased genetic variants across 12 chromosomes (N. Baig, personal communication), was randomly selected from a diverse panel of 100 clones. The genetic variants, including single‐nucleotide polymorphism and small insertion/deletion polymorphisms, were kept with a minor allele frequency > 0.05 and a missing rate < 0.1. In order to save computational time, one random marker in each 15 kb window was randomly selected to reduce the total number of markers. As a result, a total of 49,125 phased genetic variants were used in this study.

The 80 clones were used as parents of the simulated progenies at the initial breeding cycle (burn‐in cycle). The progenies were simulated via AlphaSimR (Gaynor et al., 2021). For this, the genetic map information of all genetic variants was estimated using a Marey map (Wu et al., 2023). Subsequently, the genomic information for each variant and genetic map information served as input for the simulations.

### 2.2 Breeding programs and selection strategies

This simulation study was based on three main selection strategies in a clonal potato breeding program (Figures S1 and S2): (1) Standard‐PS (a scheme following a standard potato breeding program relying exclusively on PS, which serves as benchmark, Table S1); (2) Optimal‐PS (a scheme relying on PS but where the optimal selected proportions during the selection process were determined to maximize genetic gain); (3) Optimal‐GS (a scheme based on both PS and GS where the optimal selected proportions and the optimal weight of GS relative to PS ($\alpha_{k}$) during the selection process were determined to maximize genetic gain).

To simulate a long‐term potato breeding program, 30 sequential breeding cycles were considered. Each breeding cycle of the breeding program comprised seven stages: cross stage (X), seedling stage (SL), single hills stage (SH), A clone stage (A), B clone stage (B), C clone stage (C), and D clone stage (D). During each breeding cycle, the selection was performed following one of the above‐described three selection strategies. At the end of one breeding cycle, a defined number of D clones were selected as new parents for the next breeding cycle and intercrossed to create new genetic variation. The details of the approaches used to determine new crosses are described in the next section.

**FIGURE 1:**
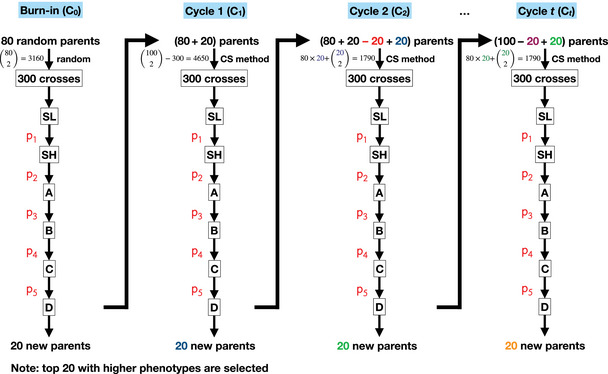
Graphical illustration of recurrent selection in a potato breeding program with the chosen cross‐selection (CS) method to determine new crosses. Each breeding cycle of the breeding program comprised seven main stages: cross stage where 300 crosses are selected, seedling stage (SL), single hills stage (SH), A clone stage (A), B clone stage (B), C clone stage (C), and D clone stage (D). $p_{1}$ to $p_{5}$ are selected proportions at each selection stage. Their exact values for each selection strategy and the details about the selection strategies in each breeding cycle are shown in Figure S1 and S2.

In order to allow for an unbiased comparison of performance across different selection strategies and CS methods, a consistent starting point, called burn‐in cycle ($C_{0}$), was required. The procedure of the potato breeding program across 30 cycles is shown in Figure 1 and its details are described in the following:

- **Burn‐in cycle** ($C_{0}$)
  – Step 1: 300 crosses were randomly selected from all possible crosses in the half‐diallel among the 80 parents (=3160, called candidate crosses) and served as a crossing plan. From each cross, the same number of progenies, which were in the following designed as SL progenies, were simulated.
  – Step 2: Selection processes from SL to D clone stages were conducted according to the chosen selection strategy (Figure S1).
  – Step 3: The top 20 of the 60 D clones were selected based on phenotypes of the target trait and were, together with the 80 parents of $C_{0}$, considered as candidate parents for cycle 1 ($C_{1}$). Therefore, the number of candidate parents in $C_{1}$ became 100 (i.e., 80 candidate parents at $C_{0}$ + 20 selected top 20 D clones of $C_{0}$).
- **Cycle 1** ($C_{1}$)
  – Step 1: Because we randomly selected 300 crosses from all possible cross combinations at $C_{0}$, we still considered all possible crosses in the half‐diallel among the 100 parents, excluding the 300 crosses used at $C_{0}$. Thus, the number of the candidate crosses at $C_{1}$ was 4650. The performance of each cross combination was then calculated based on the chosen CS method.
  – Step 2: Based on the calculated performance from Step 1, the top 300 crosses were selected as the crossing plan and, from each cross, the same number of SL progenies, were simulated.
  – Step 3: Like Step 2 of $C_{0}$.
  – Step 4: Like Step 3 of $C_{0}$ except that 20 parents were randomly removed from those candidate parents which were not used (or used only once or twice if the number of non‐used clones in the cycle was below 20) in the crossing plan of $C_{1}$. Therefore, the number of candidate parents in the next cycle ($C_{2}$) remained 100 (i.e., 80 candidate parents at $C_{0}$ + 20 selected top 20 D clones of $C_{0}$ − 20 randomly discarded if not used as parents in crossing plan $C_{1}$ + 20 selected top 20 D clones of $C_{1}$).
- **Cycle** *t* ($C_{t}$), **where** *t* > **1**
  – Step 1: To (i) mimic the breeder's approach to keep a reasonable size for candidate parents while focusing on new genotypes, and (ii) reduce computational time, only the candidate crosses which were crosses between the 80 old and 20 new ones and all possible crosses in the half‐diallel among the 20 new candidate parents were considered for $C_{t}$ and their performances were calculated according to the CS method.
  – Steps 2–4: Like Steps 2–4 of $C_{1}$.

### 2.3 Cross‐selection (CS) methods

Different methods were tested to select new crosses for the next cycle. The considered parameters for each cross were (i) the predicted progeny mean, μ; (ii) the predicted progeny variance, $\sigma_{G}^{2}$; (iii) the predicted progeny diversity; and (iv) the linear combinations of (i), (ii), and (iii).

The predicted progeny mean could be evaluated in five different ways (mean‐based CS methods): (i) the mean phenotypic values of the two parents, MPV; (ii) the mean estimated breeding values of the two parents, MEBV‐P; (iii) the mean estimated genetic values of the two parents, MEGV‐P; (iv) the mean estimated breeding values among simulated offsprings, MEBV‐O; and (v) the mean estimated genetic values among simulated offsprings, MEGV‐O. The last two, (iv) and (v), were estimated as the mean breeding and genetic values, respectively, among 1000 simulated progenies of an in silico cross. The progenies were simulated using AlphSimR.

**FIGURE 2:**
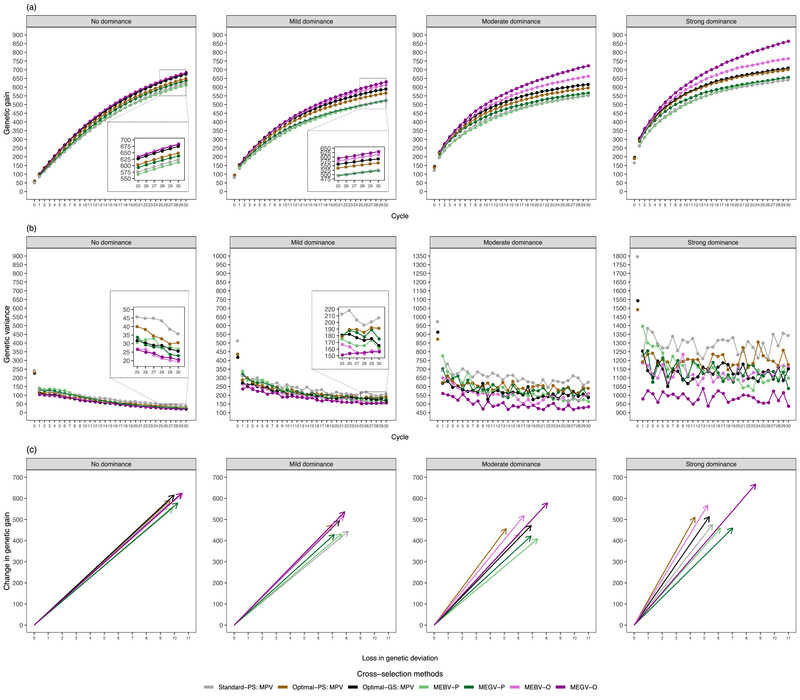
The evolution of genetic gain (a) and genetic variance (b) for the target trait along the 30 breeding cycles on average across 30 simulation runs. The efficiency of converting genetic diversity into genetic gain (c) by regressing the change of genetic gain on the loss of genetic standard deviation between cycle 0 and cycle 30. The three parameters were assessed at D clone stage for different selection strategies (Standard‐PS, Optimal‐PS, and Optimal‐GS), different mean‐based cross‐selection methods (mean phenotypic values of the two parents [MPV], mean estimated breeding values of the two parents [MEBV‐P], mean estimated genetic values of the two parents [MEGV‐P], mean estimated breeding values among simulated offspring [MEBV‐O], and mean estimated genetic values among simulated offspring [MEGV‐O]), and different genetic architectures of the target trait (no, mild, moderate, and strong dominance effects). Optimal‐GS, a scheme based on both phenotypic selection and genomic selection where the optimal selected proportions during the selection process were determined to maximize genetic gain; Optimal‐PS, a scheme relying on phenotypic selection but where the optimal selected proportions during the selection process were determined to maximize genetic gain; Standard‐PS, a scheme following a standard potato breeding program relying exclusively on phenotypic selection.

To balance the selection of new crosses between improvement of genetic gain and maintenance of variability measured by predicted progeny variance, the concept of UC (Schnell & Utz, 1975) was first extended by

(1) $$\begin{array}{c} \mathrm{EUC}:\;\mu +w_{1}\cdot i\cdot \mathrm{PA}\cdot \sigma_{G} \end{array}$$

representing an extended usefulness criterion (EUC), in which μ was the predicted progeny mean, $w_{1}$ a weight on the square root of the progeny variance ($\sigma_{G}$), i the selection intensity, and PA the prediction accuracy of the GS model. Here, PA replaced the square root of heritability in the response to selection when GS was implemented (Falconer & Mackay, 1996; Heffner et al., 2010). For EUC, μ was based on MEGV‐O because this measurement outperformed the other progeny mean estimations in our previous comparison among different mean‐based methods (Figure 2a). $\sigma_{G}^{2}$ was estimated by the variance of genetic values $T_{t}$ among 1000 simulated progenies of an in silico cross. $w_{1}$ was chosen to be either 1, 10, 50, or 100. If $w_{1}=1$, the Equation (1) is equivalent to UC. Moreover, we assumed the selected proportion per cross as 0.1 so that i corresponds to 1.755.

In addition to EUC and to keep a certain level of genomic diversity in the breeding program, a measure of the gene diversity (as the expected heterozygosity [He]) was incorporated into the Equation (1) to create an extended usefulness criterion incorporating genomic diversity index (EUCD) by:

(2) $$\begin{array}{c} \mathrm{EUCD}:\;\mu +w_{1}\cdot i\cdot \mathrm{PA}\cdot \sigma_{G}+w_{2}\cdot {\mathrm{He}}_{\mathrm{per}-\mathrm{cross}}, \end{array}$$

where $w_{1}$ was equal to 1 in EUCD in analogy to UC, ${\mathrm{He}}_{per-\mathrm{cross}}$ was used to quantify the genomic diversity of a cross and calculated as the He among 1000 simulated progenies of an in silico cross, and $w_{2}$ represented a weight on ${\mathrm{He}}_{\mathrm{per}-\mathrm{cross}}$. Due to the tetraploid nature of potato, He was determined as:

(3) $$\begin{array}{c} \mathrm{He}=\frac{1}{m}\sum_{j=1}^{m}\left(1-\sum_{i=1}^{k}p_{i(j)}^{4}\right), \end{array}$$

where m was the number of genetic variants, k the number of alleles in one genetic variant, and $p_{i(j)}$ the allele frequency of the $i^{\mathrm{th}}$ allele at the $j^{\mathrm{th}}$ genetic variant (Gallais, 2003). We only considered biallelic genetic variants in this study, and therefore, k was equal to 2.

The scale of $\sigma_{G}$ and ${\mathrm{He}}_{per-\mathrm{cross}}$ and their variance differed largely. To maintain the same level of importance for the two measurements in Equations (1) and (2), $w_{2}$ was selected to be either 50, 500, 2500, or 5000 (Table 1).

**TABLE 1:** Overview of the different weight ($w_{1}$ and $w_{2}$) scales for the extended usefulness criterion (EUC) and extended usefulness criterion incorporating genomic diversity index (EUCD), respectively.

Criterion	Cross‐selection 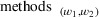			
	Scale A	Scale B	Scale C	Scale D
EUC	${\mathrm{EUC}}_{\left(1,0\right)}$	${\mathrm{EUC}}_{\left(10,0\right)}$	${\mathrm{EUC}}_{\left(50,0\right)}$	${\mathrm{EUC}}_{\left(100,0\right)}$
EUCD	${\mathrm{EUCD}}_{\left(1,50\right)}$	${\mathrm{EUCD}}_{\left(1,500\right)}$	${\mathrm{EUCD}}_{\left(1,2500\right)}$	${\mathrm{EUCD}}_{\left(1,5000\right)}$

*Note*: $w_{1}$ is a weight on the square root of the progeny variance, and $w_{2}$ a weight on genome‐wide diversity quantified by expected heterozygosity (He).

### 2.4 Simulation of genetic architecture of traits

#### 2.4.1 Simulated true genetic and phenotypic values

Two traits, auxiliary ($T_{a}$) and target ($T_{t}$) traits, were considered in this study. Here, $T_{a}$ represented the weighted sum of the auxiliary traits measured in the first three stages of the breeding program, and $T_{t}$ the weighted sum of all market‐relevant quantitative traits. The latter was controlled by 2000 quantitative trait loci (QTLs). Each QTL included additive and dominance effects, and had five possible genotype classes: aaaa, Aaaa, AAaa, AAAa, and AAAA. The true additive effects of 2000 QTLs were drawn from a gamma distribution with k=2 and θ=0.2, where k and θ are shape and scale parameters, respectively. The dominance effects, being the deviation of genetic value from the breeding value, were set differently for the three heterozygous genotypes (Aaaa, AAaa, and AAAa) and expressed by $d_{1}$, $d_{2}$, and $d_{3}$, respectively (Table 2; Gallais, 2003). For each QTL, the degree of dominance (ratio of dominance to additive effect δ) was produced from a normal distribution N(1,1) (cf. Werner et al., 2023). The true dominance effect at each QTL was then calculated by multiplying the true additive effect by the QTL‐specific δ. Finally, the true genetic value (TGV) for $T_{t}$ was calculated for each clone by summing up the true additive and dominance effects across 2000 QTLs. The TGV for $T_{a}$ was controlled by the genetic correlations between $T_{a}$ and $T_{t}$ (r). The details of the simulated ${\mathrm{TGV}}_{T_{a}}$ were described in Method S1.

**TABLE 2:** Summary of the five genotype classes, including their coding expression, additive and dominance effects, as well as breeding and genetic values.

Genotype class	Additive effect (a)	Dominance effect			Breeding value	Genetic value
		$d_{1}$	$d_{2}$	$d_{3}$		
aaaa	0	0	0	0	0	0
Aaaa	1	1	0	0	a	a + $d_{1}$
AAaa	2	0	1	0	2a	2a + $d_{2}$
AAAa	3	0	0	1	3a	3a + $d_{3}$
AAAA	4	0	0	0	4a	4a

The phenotypic values (P) were calculated as P=TGV+ε, where ε was a non‐genetic value following a normal distribution $N(0,\sigma_{\varepsilon }^{2})$, in which $\sigma_{\varepsilon }^{2}$ was the non‐genetic variance. Non‐genetic variance for $T_{t}$ ($\sigma_{\varepsilon_{T_{t}}}^{2}$) was determined by the following equation:

(4) $$\sigma_{\varepsilon_{T_{t}}}^{2}=\frac{\sigma_{G_{T_{t}}\times L}^{2}}{L_{j}}+\frac{\sigma_{{\mathrm{trial}}_{T_{t}}}^{2}}{L_{j}},$$

where $\sigma_{G_{T_{t}}\times L}^{2}$ was the variance of interaction between genotype and location, $\sigma_{{\mathrm{trial}}_{T_{t}}}^{2}$ the trial error variance, and $L_{j}$ the number of location at stage j, where j∈{B,C,D} (Table S1). Non‐genetic variance for $T_{a}$ ($\sigma_{\varepsilon_{T_{a}}}^{2}$) (=trial error variance, $\sigma_{{\mathrm{trial}}_{T_{a}}}^{2}$) was determined by the following equation:

(5) $$\sigma_{\varepsilon_{T_{a}}}^{2}=\frac{1-H_{T_{a}}^{2}}{H_{T_{a}}^{2}}\sigma_{G_{T_{a}}}^{2}.$$

In this study, the trial environments across locations and breeding cycles were assumed to be homogeneous, and therefore $\sigma_{{\mathrm{trial}}_{T_{a}}}^{2}$ and $\sigma_{{\mathrm{trial}}_{T_{t}}}^{2}$ were fixed. To do so, $\sigma_{{\mathrm{trial}}_{T_{a}}}^{2}$ and $\sigma_{{\mathrm{trial}}_{T_{t}}}^{2}$ were estimated at SL of $C_{0}$ and were then assumed fixed for the following cycles. In detail, the ratio of variance components was set for $T_{t}$ as follows: $\sigma_{G_{T_{t}}}^{2}:\sigma_{G_{T_{t}}\times L}^{2}:\sigma_{\varepsilon_{{\mathrm{trial}}_{T_{t}}}}^{2}=1:1:0.5$, and the corresponding heritability ($H_{T_{t}}^{2}$) at each breeding stage was calculated as $H_{T_{t}}^{2}=\frac{\sigma_{G_{T_{t}}}^{2}}{\sigma_{G_{T_{t}}}^{2}+\sigma_{\varepsilon_{T_{t}}}^{2}}$. For instance, the $H_{T_{t}}^{2}$ at D clone stage was 0.73. The heritability of $T_{a}$ ($H_{T_{a}}^{2}$) was fixed to 0.6. At SL of $C_{0}$, $\sigma_{G_{T_{a}}}^{2}$ and $\sigma_{G_{T_{t}}}^{2}$ were estimated by the sample variance of ${\mathrm{TGV}}_{T_{a}}$ and ${\mathrm{TGV}}_{T_{t}}$, respectively. Then, $\sigma_{\varepsilon_{{\mathrm{trial}}_{T_{t}}}}^{2}$ was fixed to $\frac{1}{2}$ of the estimated $\sigma_{G_{T_{t}}}^{2}$. Similarly, $\sigma_{\varepsilon_{{\mathrm{trial}}_{T_{a}}}}^{2}$ was estimated by Equation (5). However, $\sigma_{G_{T_{t}}}^{2}$ and $\sigma_{G_{T_{t}}\times L}^{2}$ varied across breeding cycles and $\sigma_{G_{T_{t}}}^{2}$ was re‐estimated at SL of each cycle. Consequently, $\sigma_{G_{T_{t}}\times L}^{2}$ was controlled by the ratio of variance components.

#### 2.4.2 Estimated breeding and genetic values

In this study, a GS model was assumed to be trained for $T_{t}$ at earlier cycles of the breeding program and was updated regularly to maintain a relatively high and consistent degree of prediction accuracy (PA). The estimated breeding values for $T_{t}$ obtained from the GS model were estimated by ${\mathrm{EBV}}_{T_{t}}={\mathrm{TBV}}_{T_{t}}+\varepsilon_{\mathrm{PA}}$, where ${\mathrm{TBV}}_{T_{t}}$ were the true breeding values of $T_{t}$, for which only additive effects were considered. $\varepsilon_{\mathrm{PA}}$ was the residual value following a normal distribution $N(0,\sigma_{\varepsilon_{\mathrm{PA}}}^{2})$, with

(6) $$\begin{array}{c} \sigma_{\varepsilon_{\mathrm{PA}}}^{2}=\frac{1}{n^{\prime }-2}\frac{1-{\mathrm{PA}}^{2}}{{\mathrm{PA}}^{2}}\sum_{i=1}^{n^{\prime }}({\mathrm{TBV}}_{T_{t}\left(i\right)}-{\bar{\mathrm{TBV}}}_{T_{t}})2 \end{array}$$

representing the error variance determined by the level of PA, where $n^{\prime }$ was the number of genotyped clones, ${\mathrm{TBV}}_{T_{t}\left(i\right)}$ the ${\mathrm{TBV}}_{T_{t}}$ at the $i^{\mathrm{th}}$ genotyped clone, and ${\bar{\mathrm{TBV}}}_{T_{t}}$ the average of ${\mathrm{TBV}}_{T_{t}}$ on all genotyped clones. The estimated genetic values for $T_{t}$ (${\mathrm{EGV}}_{T_{t}}$) were obtained by replacing all TBV appearing in this section by TGV.

### 2.5 Economic settings and quantitative genetic parameters

The costs for phenotypic evaluation of $T_{a}$ and $T_{t}$ in one environment were assumed to be 1.4 and 25 €, respectively. The costs for genotypic evaluation per clone were set to 25 €. For the Standard‐PS procedure (Table S1), the total budget in one breeding cycle was 677,500 €. As this strategy served as benchmark, the total budget for all other selection strategies was also fixed to this amount. In a previous study, the selection strategy GS‐SH:A under optimal selected proportions achieved the maximum short‐term genetic gain (Wu et al., 2023). Thus, we chose the selection strategy GS‐SH:A as Optimal‐GS in this study, and set PA and r to 0.5 and 0.15, respectively, for all selection strategies as well as CS methods. The same number of locations and number of clones at D ($N_{6}$ = 60) were set as the ones in the Standard‐PS. The optimal selected proportions ($p_{i}$ and $\alpha_{k}$ if integration of GS) achieving the maximum short‐term genetic gain were based on the results in Wu et al. (2023) (see for details Method S2). The optimal selected proportions ($p_{i}$ and $\alpha_{k}$ if integration of GS) and the number of clones at SL for each selection strategy used in this study are summarized in Figure S1.

In order to investigate the interaction effects between different genetic architectures and CS methods on long‐term genetic gain, we considered four different cases of δ for $T_{t}$: (1) No dominance effects: $\delta_{0}$ was set to 0; (2) mild dominance effects: $\delta_{1}$ was produced across all QTLs from N(1,1) as abovementioned; (3) moderate dominance effects: $\delta_{2}=2\times \delta_{1}$; and (4) strong dominance effects: $\delta_{3}=3\times \delta_{1}$.

### 2.6 Evaluations

The genetic gain and genetic variability of ${\mathrm{TGV}}_{T_{t}}$, the genome‐wide diversity, as well as the number of QTLs where the favorable allele was fixed or lost were estimated and ranked for each scenario in each breeding cycle. The genetic gain was defined as the difference in mean ${\mathrm{TGV}}_{T_{t}}$ between progenies at D clone stage and the 80 selected candidate parents of $C_{0}$. The level of variability was evaluated by the genetic variance of $T_{t}$, and the level of genomic diversity by the He (see Equation 3) at D clone stage. The number of QTLs where the favorable allele was fixed (=all progenies carrying genotype with AAAA) or lost (=all progenies carrying genotype with aaaa) was calculated among the progenies at D clone stage. To avoid effects due to sampling, all results in this study were based on 30 independent simulation runs.

The efficiency of converting genetic diversity into genetic gain was measured by regressing the realized genetic gain (y) on the loss of genetic diversity (x), that is, y=a+bx+e, in which the slope (b) was efficiency (Gorjanc et al., 2018). In this study, large fluctuations in genetic variance were noticed especially with increased dominance effects. Thus, the realized genetic gain (y) was calculated by the difference in averaged genetic gain among 30 simulation runs between $C_{0}$ and $C_{30}$. Similarly, the loss of genetic diversity was computed as the difference in the averaged genetic standard deviation among 30 simulation runs between $C_{0}$ and $C_{30}$.

To assess the accuracy in predicting progeny mean using different mean‐based CS methods, we calculated the real progeny mean as the average of ${\mathrm{TGV}}_{T_{t}}$ of all simulated SL progenies at $C_{0}$ and $C_{30}$, respectively. The prediction accuracy was estimated as the correlation between real and predicted progeny mean on an average across 30 simulation runs.

## 3 RESULTS

The mean genetic gain and genetic variance of $T_{t}$, the efficiency of converting genetic diversity into genetic gain, the genome‐wide diversity, as well as the number of QTLs where the favorable allele was fixed or lost in a long‐term tetraploid potato breeding program were assessed considering the following parameters and their interactions: (1) different selection strategies, (2) different CS methods, and (3) different genetic architectures of $T_{t}$, that is, different degree of dominance. A total of 30 simulation runs were performed for these assessments. To check for a bias between the CS methods, the standard deviation for the genetic gain among 30 runs was compared. The homogeneous standard deviation found across the CS methods (data not shown) ensured a meaningful comparison hereafter.

Regardless of the genetic architectures of $T_{t}$ and using the MPV method, any selection strategy based on the optimal allocation of resources (Optimal‐GS and Optimal‐PS) had a higher genetic gain than the Standard‐PS in both short‐ and long‐term breeding programs (Figure 2a). Furthermore, Optimal‐GS was superior to Optimal‐PS. An increase in the cycle numbers strengthened this tendency.

Regardless of the selection strategies, CS methods, and genetic architectures of $T_{t}$, improved genetic gain was observed with an increased number of completed breeding cycles (Figures 2a and 5a). However, the additional genetic gain per cycle became smaller at late breeding cycles compared to early ones. This trend as well as the difference in ranking among all assessed CS methods were affected by several parameters: the degree of dominance and weights ($w_{1}$ and $w_{2}$) of the modified UC. The details thereof are explained below.

**FIGURE 3:**
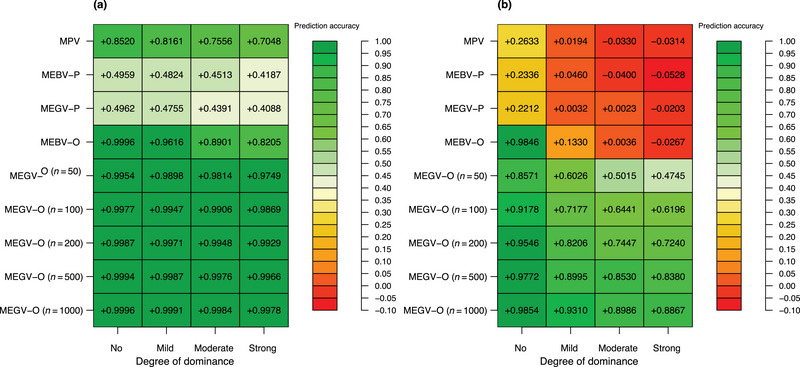
Accuracy to predict progeny mean using the different mean‐based cross‐selection methods (mean phenotypic values of the two parents [MPV], mean estimated breeding values of the two parents [MEBV‐P], mean estimated genetic values of the two parents [MEGV‐P], mean estimated breeding values among simulated offspring [MEBV‐O], and mean estimated genetic values among simulated offspring [MEGV‐O]) under different genetic architectures of the target trait. The accuracy was calculated as the correlation between predicted progeny mean and real progeny mean at seedling stage of $C_{0}$ (a) and $C_{30}$ (b), respectively on an average across 30 simulation runs. To examine whether the population size of simulated progenies affects the prediction accuracy using MEGV‐O, five different population sizes of the simulated progeny (n = 50, 100, 200, 500, and 1000) were considered.

**FIGURE 4:**
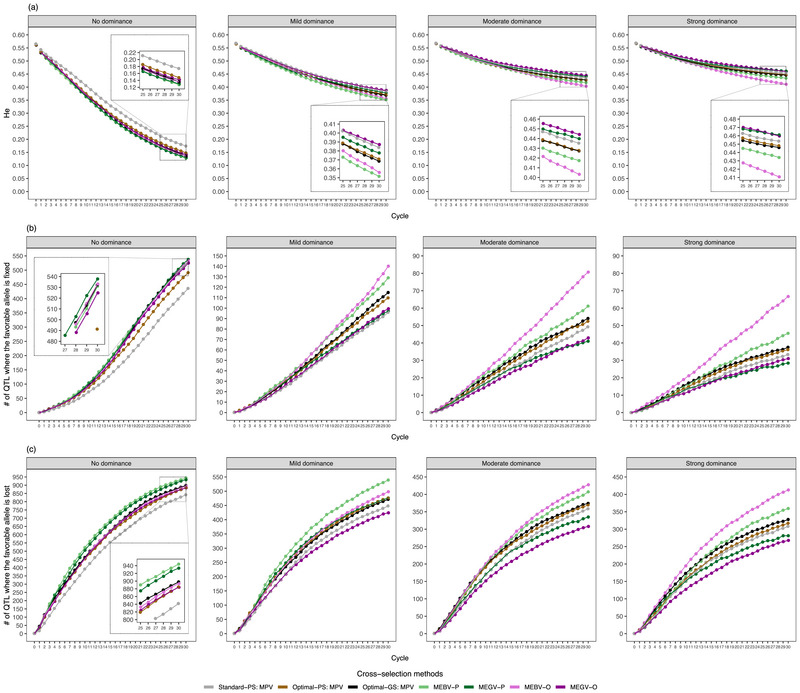
The evolution of genome‐wide diversity measured by expected heterozygosity (He) (a), number of quantitative trait loci (QTLs) where the favorable allele is fixed (b) and lost (c), along the 30 breeding cycles on average across 30 simulation runs. The three parameters were assessed at D clone stage for different selection strategies (Standard‐PS, Optimal‐PS, and Optimal‐GS), different mean‐based cross‐selection methods (mean phenotypic values of the two parents [MPV], mean estimated breeding values of the two parents [MEBV‐P], mean estimated genetic values of the two parents [MEGV‐P], mean estimated breeding values among simulated offspring [MEBV‐O], and mean estimated genetic values among simulated offspring [MEGV‐O]), and different genetic architectures of the target trait (no, mild, moderate, and strong dominance effects). Optimal‐GS, a scheme based on both phenotypic selection and genomic selection where the optimal selected proportions during the selection process were determined to maximize genetic gain; Optimal‐PS, a scheme relying on phenotypic selection but where the optimal selected proportions during the selection process were determined to maximize genetic gain; Standard‐PS, a scheme following a standard potato breeding program relying exclusively on phenotypic selection.

**FIGURE 5:**
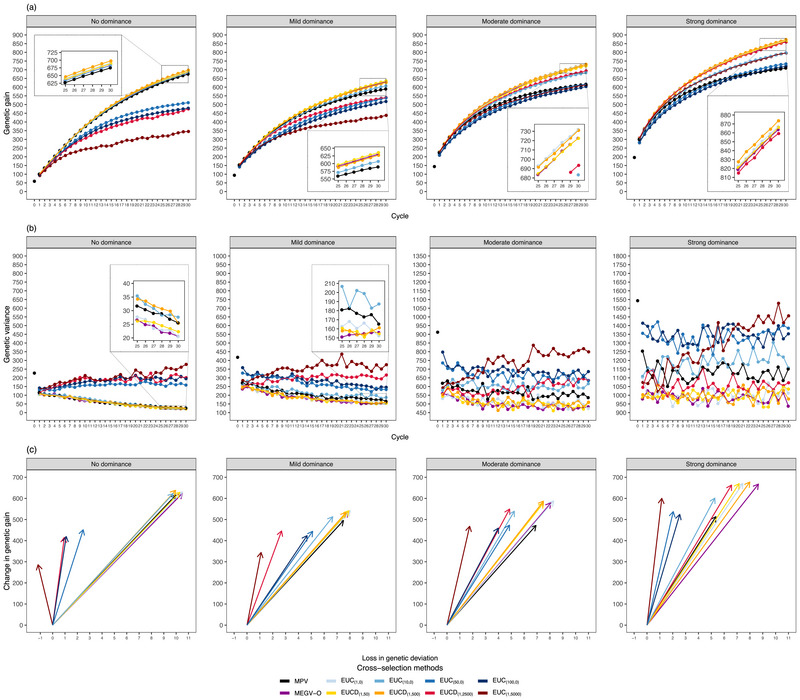
The evolution of genetic gain (a) and genetic variance (b) for the target trait along the 30 breeding cycles on average across 30 simulation runs. The efficiency of converting genetic diversity into genetic gain (c) by regressing the change of genetic gain on the loss of genetic standard deviation between cycle 0 and cycle 30. The three parameters were assessed at D clone stage based on Optimal‐GS (a scheme based on both phenotypic selection and genomic selection where the optimal selected proportions during the selection process were determined to maximize genetic gain) selection strategy for different cross‐selection methods modified by usefulness criteria (extended usefulness criterion [EUC] and extended usefulness criterion incorporating genomic diversity index [EUCD]), and different genetic architectures of the target trait (no, mild, moderate, and strong dominance effects). The details of EUC and EUCD are shown in Table 1. MEGV‐O, mean estimated genetic values among simulated offspring; MPV, mean phenotypic values of the two parents.

### 3.1 Comparison of CS methods that only consider progeny mean

First, we evaluated the effects of the implementation of GS on genetic gain using different CS methods only focusing on the progeny mean. In general, any progeny mean predicted by in silico progenies (MEBV‐O and MEGV‐O) outperformed those predicted by mid‐parental performance (MPV, MEBV‐P, and MEGV‐P) (Figure 2a). Furthermore, the MEGV‐O method was superior to the MEBV‐O method. The difference between these two CS methods became more obvious with increasing numbers of breeding cycles as well as an increased degree of dominance. The dominance level showed a stronger influence on genetic gain than the cycle numbers. In addition, the MPV (Optimal‐GS) had the highest long‐term genetic gain among CS methods based on mid‐parental performance. Interestingly, a higher prediction accuracy in predicting progeny mean was observed for the methods based on in silico progenies compared to those based on mid‐parental performance (Figure 3).

In contrast to the genetic gain, the genetic variance of $T_{t}$ decreased as the number of breeding cycles increased (Figure 2b). This tendency increased with the reduction of the degree of dominance. Furthermore, the effects of the selection strategies and the CS methods on the genetic variance were in opposition to the one on the genetic gain (Figure 2a,b). As the degree of dominance increased, larger differences and fluctuations in genetic variance among these CS methods and across cycles were observed.

On the other hand, all mean‐based CS methods had similar efficiency of converting genetic diversity into genetic gain under the cases without and with low dominance effects (Figure 2c). With increasing dominance effects, MEGV‐O did not reach the largest efficiency among all mean‐based CS methods. However, its genetic gain was about 1.3 times higher than the CS method achieving the highest efficiency under Optimal‐PS.

With increasing numbers of completed breeding cycles, the genome‐wide diversity measured as He decreased (Figure 4a). Simultaneously, the number of QTLs where the favorable allele was fixed or lost also increased (Figure 4b,c). However, a higher degree of dominance reduced this tendency. With an increase in the importance of dominance effects, the CS methods considering additive and dominance effects (MPV, MEGV‐P, and MEGV‐O) maintained a higher He and resulted in a lower number of fixed QTLs than those based solely on additive effects (MEBV‐P and MEBV‐O), especially at late cycles. Furthermore, the MEGV‐O method maintained the highest He and had the lowest number of fixed QTLs among the progeny mean‐based CS methods, even though it had the lowest genetic variance and the highest genetic gain. Therefore, MEGV‐O was used hereafter as the measurement for the prediction of progeny mean in the weighted methods, that is, EUC and EUCD.

### 3.2 Comparison of CS methods with weights on progeny variance or genome‐wide diversity

Regardless of the genetic architecture of $T_{t}$, a small or no difference in genetic gain was observed at early cycles among the following CS methods: MEGV‐O, EUC, and EUCD with low weights (Figure 5a). As the cycle number increased, the difference became more pronounced. On average across the four levels of dominance effects, ${\mathrm{EUC}}_{\left(1,0\right)}$ (=UC) had the highest genetic gain among all EUC approaches (731.01 at $C_{30}$) and was superior to CS methods based only on progeny mean (MEGV‐O and MPV methods) (Figure 5a, Table S2). Furthermore, EUCD with a low weight ($w_{2}$ = 50 or 500) yielded the highest genetic gain (734.38 at $C_{30}$).

We compared four different levels of importance for the variability aspect (being genetic variance or He) in EUC/EUCD on the long‐term gain of selection. These were called Scale A, B, C, and D (Table 1). Regardless of the genetic architecture of $T_{t}$, no significant difference between the genetic gain of EUCD and EUC was observed when the lowest weights for $w_{1}$ and $w_{2}$ were considered (i.e., Scale A, Figure 5a, Table S2). Furthermore, ${\mathrm{EUCD}}_{\left(1,500\right)}$ always outperformed ${\mathrm{EUC}}_{\left(10,0\right)}$ (Scale B). With high dominance effects, the EUCDs were superior to the EUCs with high weights, that is, under Scale C and D.

The ranking and the difference in genetic gain among the abovementioned CS methods were influenced by the degree of dominance (Table S2). EUC and EUCD with high weights ranked better with increasing contributions from dominance effects. This was especially true for EUCD. For instance, ${\mathrm{EUCD}}_{\left(1,5000\right)}$ had the worst performance under no or mild dominance effects. However, with strong dominance effects, it ranked seventh and outperformed ${\mathrm{EUC}}_{\left(50\&100,0\right)}$, as well as MPV. While a slow improvement of genetic gain using ${\mathrm{EUCD}}_{\left(1,2500\right)}$ was observed under the case without dominance effects, it ranked fifth under the cases with moderate and strong dominance effects. Furthermore, the difference between this CS method and the best one decreased, especially in the case of strong dominance effects.

EUC and EUCD with low weights resulted in high genetic gain but reduced genetic variance (Figure 5a,b, Table S2). This trend was similar to the mean‐based CS methods described in the previous section. In addition, with an increase in cycle numbers, the reduction of genetic variance slowed down, especially for the scenario with strong dominance effects. By contrast, high‐weighted EUC and EUCD maintained relatively high genetic variance and even increased it as the cycle number increased.

The CS methods were also evaluated for their effects on genetic variance within each scale (Table 1). EUCD resulted also in a higher genetic variance than EUC under Scale C and D, except for the case with strong dominance effects under Scale C. Nevertheless, EUCD resulted in a higher genetic gain than EUC. Furthermore, with strong dominance effects, ${\mathrm{EUCD}}_{\left(1,5000\right)}$ maintained the highest genetic variance. However, it still performed similarly to ${\mathrm{EUC}}_{\left(10,0\right)}$ regarding genetic gain and even had much higher genetic gain than MPV and ${\mathrm{EUC}}_{\left(50\&100,0\right)}$.

In general, any EUC and EUCD had higher efficiency of converting genetic diversity into genetic gain than MEGV‐O, especially with increasing importance of dominance effects (Figure 5c). Higher weights for EUC and especially EUCD had a higher efficiency but lower genetic gain compared to lower weights. By contrast, as the importance of dominance effects increased, the difference in genetic gain gradually diminished between using CS methods with higher weights (still reaching a higher efficiency) and CS methods with lower weights.

**FIGURE 6:**
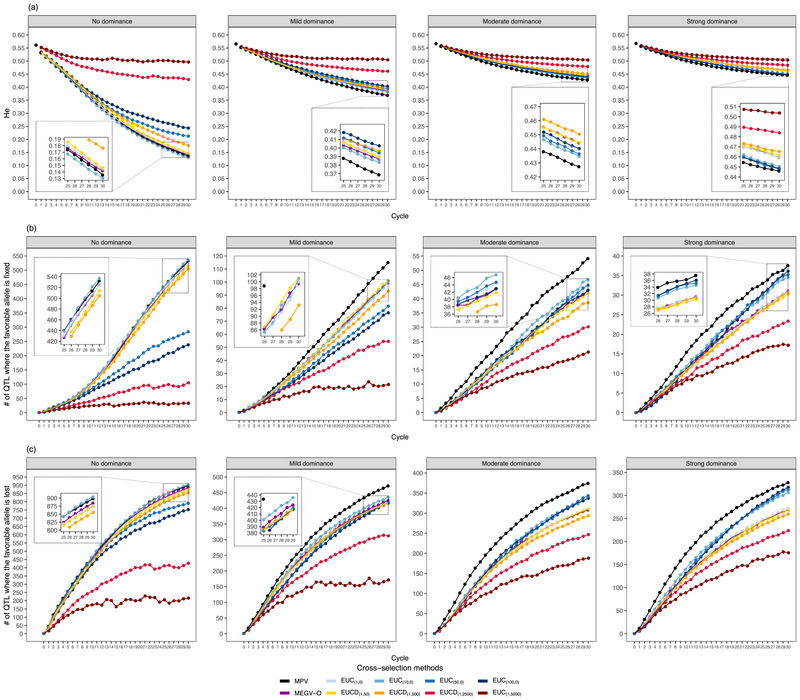
The evolution of genome‐wide diversity measured by expected heterozygosity (He) (a), number of quantitative trait loci (QTLs) where the favorable allele is fixed (b) and lost (c), along the 30 breeding cycles on average across 30 simulation runs. The three parameters were assessed at D clone stage based on the Optimal‐GS (a scheme based on both phenotypic selection and genomic selection where the optimal selected proportions during the selection process were determined to maximize genetic gain) selection strategy for different cross‐selection methods modified by usefulness criteria (extended usefulness criterion [EUC] and extended usefulness criterion incorporating genomic diversity index [EUCD]), and different genetic architectures of the target trait (no, mild, moderate, and strong dominance effects). The details of EUC and EUCD are shown in Table 1. MEGV‐O, mean estimated genetic values among simulated offspring; MPV, mean phenotypic values of the two parents.

On the other hand, along increasing cycles, EUC dramatically decreased He and increased the number of QTLs where the favorable allele was fixed or lost (Figure 6, Table S2) and, thus, had similar trends to the mean‐based CS methods. These trends were not substantially mitigated as $w_{1}$ increased, except for the scenarios with low or no dominance effects. In contrast to EUC, using EUCD obviously slowed down the decline of He, and simultaneously reduced the number of fixed QTLs. A greater $w_{2}$ increased this tendency.

EUCD maintained a higher He and a lower number of fixed QTLs than EUC for each scale (Figure 6). Furthermore, EUCD with a low $w_{2}$ reached a higher He and resulted in a lower number of fixed QTLs compared to EUC with a high $w_{1}$, especially when dominance effects had a high importance (Figure 6, Table S2). Across all genetic architectures of $T_{t}$, EUCD with a low $w_{2}$ (50 or 500) achieved high genetic gain and still maintained a higher He and a lower number of fixed QTLs than the UC and the MEGV‐O method (Figure 6). Meanwhile, EUCD's level of genetic variance remained average. Under strong dominance effects, the genetic gain realized by EUCD with a high $w_{2}$ (e.g., ${\mathrm{EUCD}}_{\left(1,2500\right)}$) had no significant difference compared to the highest one achieved by ${\mathrm{EUCD}}_{\left(1,500\right)}$ (Table S2). However, higher He, genetic variance, and efficiency of converting genetic diversity into genetic gain and a lower number of fixed QTLs were achieved by ${\mathrm{EUCD}}_{\left(1,2500\right)}$ compared to the ones realized by ${\mathrm{EUCD}}_{\left(1,500\right)}$ (Figures 5 and 6).

## 4 DISCUSSION

The effects of CS methods and the incorporation of genetic diversity have been evaluated in diploid crops to enhance genetic gain (Allier et al., 2019; Gaynor et al., 2017; Werner et al., 2023). However, the effects of implementing GS in different CS methods on long‐term genetic gain for autotetraploid crops with a highly heterozygous genome are lacking. Because of their difference in quantitative genetics compared to diploid inbred or hybrid systems, one might expect different outcomes in such analyses. Therefore, we evaluated the efficiency of different CS methods in long‐term breeding programs under different genetic architectures via a simulation study.

### 4.1 The effects of different selection strategies on long‐term potato breeding programs

In this study, we extended the study of GS efficiency from short‐term (Wu et al., 2023) to long‐term genetic gain. Regardless of the genetic architectures and based on MPV as CS method, a higher genetic gain (Figure 2a) was observed in long‐term breeding programs with Optimal‐PS compared to the benchmark Standard‐PS. This follows the trend observed in the study on short‐term genetic gain (Wu et al., 2023). The reason is that Optimal‐PS had lower selected proportions at B and C clone stages (i.e., higher selection intensities, Figure S1), which were fully based on $P_{T_{t}}$ selection in comparison to the benchmark procedure. This in turn leads to higher genetic gain according to the breeder's equation (Falconer & Mackay, 1996). Furthermore, the selection strategy incorporating GS reached a higher genetic gain than PS did, which can be expected because the former has a higher indirect selection response than the latter at the early stages (Wu et al., 2023). Thus, we compared in the following the performance of the evaluated CS methods using the selection strategy GS‐SH:A, that is, GS was applied at SH and A.

### 4.2 The accuracy of predicting progeny mean

Among the examined mean‐based CS methods, the ranking with respect to the maximum genetic gain was MEGV‐O > MEBV‐O > MPV > MEGV‐P and MEBV‐P (Figure 2a). This trend was even more pronounced with an increasing number of breeding cycles and an increasing degree of dominance. One reason might be that the CS methods that rely on simulated offspring can more precisely predict progeny mean compared to mid‐parental performance incorporating GS (MEBV‐P and MEGV‐P) because the former allows for the estimation of the allele effects more precisely across the progenies of a cross compared to deriving it from parental information. The accuracy of predicting progeny mean (Figure 3) was in complete agreement with our finding about the ranking of the CS methods with respect to their genetic gain. In addition, to examine whether the population size of the simulated progenies affects the degree of prediction accuracy, we varied the number of simulated progeny (n = 50, 100, 200, 500, and 1000). The prediction accuracies among different population sizes of simulated progeny varied only marginally compared to the ones among different CS methods. Thus, CS methods based on simulated offsprings of a cross result in higher genetic gain compared to CS methods based on mid‐parental values (Figure 2a).

Outbred crops have a highly heterozygous genome, which is accompanied by the importance of dominance effects for quantitative traits. However, the proportion of dominance variance components in total genetic variance (including additive and dominance effects) varies depending on the assessed traits and breeding materials. For instance, Endelman et al. (2018) showed that in tetraploid potato dominance variance accounted for 9.4%, 13.3%, and 16.4% of the total genetic variance for the traits specific gravity, yield, and fry color, respectively. In contrast, K. Thelen (personal communication) showed that dominance variance explained between 0% and 81.1% of the genetic variance for various agronomic traits. For example, they reported a dominance variance of 50% for tuber yield, which is considerably higher than the one reported by Endelman et al. (2018). On the other hand, the dominance effects in heterozygous species can be partially transmitted from parents to progenies (Endelman et al., 2018; Gallais, 2003; Werner et al., 2023; Wolfe et al., 2021). Therefore, taking into account dominance effects to predict progeny mean can lead to more accurate estimates compared to additive effects only. This was clearly observed in our results based on tetraploid potato: MEGV‐O had higher accuracy in predicting progeny mean compared to MEBV‐O, especially as the importance of dominance effects increased (Figure 3). It also provided higher long‐term genetic gain, which is in accordance with a previous study (Werner et al., 2023). These authors showed that genetic gain increased when considering both additive and dominance effects to predict cross performance using a formula in a diploid crop. However, our previous statement about the superiority of methods incorporating dominance effects to predict progeny mean was in discordance with our observation that MEGV‐P's genetic gain did not outperform MEBV‐P's genetic gain, despite the fact that only MEGV‐P considered dominance effects. One explanation might be that using MEGV‐P based on parental dominance effects to capture dominance effects for progenies is an incorrect assumption, leading to a low accuracy in predicting progeny mean, especially with increasing dominance effects (Figure 3).

One surprising aspect was that MPV had the highest genetic gain among all CS methods that use mid‐parental performance. This observation that phenotypic selection outperformed estimated values from a GS model was unexpected and stood in contrast to studies in maize breeding programs (Allier et al., 2019; Sanchez et al., 2023), where MEBV‐P reached a higher genetic gain than MPV. One explanation of the superiority of MPV compared to MEBV‐P and MEGV‐P in our study is that the heritability across the four environments (0.73 at D clone stage of $C_{0}$) used in the first method was higher than the assigned PA (0.5) used in the latter ones. Therefore, according to the breeder's equation, the MPV can increase the genetic gain more than other CS methods based on mid‐parental performance incorporating a GS model. This result was also confirmed by the observed higher accuracy in predicting progeny mean using MPV compared to MEBV‐P and MEGV‐P (Figure 3).

### 4.3 Limitations of mean‐based CS methods

Besides genetic gain, the evaluation of genetic variability across cycles is essential because low genetic variations in breeding materials could limit genetic gain in the long term (Falconer & Mackay, 1996). As expected, both the genetic variance of $T_{t}$ and He decreased with increasing cycle numbers (Figures 2b and 4a). At the same time, the number of QTLs where the favorable allele was fixed or lost also increased (Figure 4). The reduction in genetic variance was more pronounced especially for the CS methods achieving higher genetic gain. The high accuracy in predicting progeny mean, which leads to the quick accumulation of favorable alleles (Figure 4b), might be one reason for this observation. Moreover, the Bulmer effect (Bulmer, 1971), which reduces the proportion of genetic variance due to linkage disequilibrium between trait‐coding polymorphisms (Van Grevenhof et al., 2012), may further explain this result. In order to assess the potential importance of the Bulmer effect, we calculated the maximum genetic gain as the difference between the maximum genetic value and mean genetic values among the 80 selected candidate parents of $C_{0}$, where the maximum genetic value was obtained by summing up the maximum genetic values among the five genotypes of each QTL (Table 2) across the 2000 QTLs. The genetic gain of the mean‐based CS methods gradually closed up to the maximum genetic gain under the case without dominance effects (Figure S3), implying that the influence of the Bulmer effect was not high.

Overall, only focusing on mean performance to select new crosses could lead to a plateau for genetic gain with increasing cycle numbers. Therefore, CS methods considering the maintenance of diversity while maximizing long‐term genetic gain are required.

### 4.4 The efficiency of CS methods for balancing genetic gain and maintenance of diversity

Besides high progeny mean, a high variance in progenies is also important for the response to selection. The UC of a cross considers these aspects and has been used to predict the mean performance of the upper fraction of its progeny, considering the genetic variance, the heritability, as well as the selection intensity (Allier et al., 2019; Sanchez et al., 2023). Thus, this method could improve the genetic gain compared to mean‐based CS methods, which is confirmed in our study (Figure 5a, Table S2). While we observed slightly higher genetic gain using the UC compared to the MEGV‐O method (Figure 5, Table S2), the genetic variance and He were the same for UC and MEGV‐O method. Furthermore, the difference in genetic gain between UC and MEGV‐O was not statistically significant, which is contradictory to the results of former studies in diploid crops (Lehermeier et al., 2017; Sanchez et al., 2023). This could be explained by the lower PA (0.5) and selection intensity (1.75) used in the present study, compared to a high heritability (1) and selection intensity (2.06) in Sanchez et al. (2023). Lehermeier et al. (2017) also showed that higher heritability and selection intensity lead to a higher advantage of the UC versus other methods.

On the other hand, the variance in the progeny mean was much higher (∼90 times) than the variance in the progeny standard deviation in our study. This is in accordance with former studies (Lado et al., 2017; Zhong & Jannink, 2007), leading to no difference between UC and progeny mean. Thus, one way to strengthen the importance of the genetic variance in the progeny could be to increase the weight of the genetic variance or to add an extra variation measurement to the UC.

The genetic diversity of a cross can be quantified by the genetic variance of a trait, but also on a genome‐wide scale by the He estimated from molecular genetic information. Therefore, in addition to the weight on genetic variance of $T_{t}$, that is, EUC, one could consider weighting He to integrate another level of diversity to balance genetic gain. This is because the latter considers the level of total genomic variation instead of being restricted to the variation of specific loci linked to QTLs of $T_{t}$ like the former. In our study, on average across the four different genetic architectures (from no to strong dominance), ${\mathrm{EUCD}}_{\left(1,50|500\right)}$ reached the maximum genetic gain among all assessed EUCDs and slightly higher long‐term genetic gain compared to UC (Figure 5a, Table S2). Meanwhile, ${\mathrm{EUCD}}_{\left(1,50|500\right)}$ maintained a certain degree of genetic variance, a slightly higher He, as well as a somewhat lower number of fixed QTLs compared to UC (Figures 5 and 6). This confirmed our expectation, as EUCD maintains the advantage of the UC and preserves a certain genome‐wide diversity by accounting for He simultaneously, which in turn helps to efficiently convert genetic variability into long‐term genetic gain (Figure 5c).

Compared to low weight, EUCD with a high weight maintained higher genetic variance, He, and fewer fixed QTLs along the cycles but reduced the long‐term genetic gain. This was not surprising because a high weight on diversity means minimizing the loss of diversity after selection. Allier et al. (2019) adopted a similar approach, using weighted penalties on He to balance between maximizing genetic gain and minimizing diversity loss during cross selection. They found that stronger penalties on diversity‐limited genetic gain improvement but preserved higher diversity levels.

However, this trend of lower genetic gain with a higher $w_{2}$ gradually diminished as the degree of dominance increased in our study, implying different weights should be fitted to different genetic architectures when using EUCD, as different degrees of dominance appear in the agronomic traits of potato in experimental studies.

Although our proposed method EUCD does not achieve a significant improvement in genetic gain compared to EUC and MEGV‐O, it maintains a higher genome‐wide diversity, which can balance maximal genetic gain and minimal loss of diversity in the process of selecting new crosses. Preserving diversity is very important in long‐term breeding programs because it provides opportunities for breeders to promptly adjust the goals of the breeding programs in response to new requests such as changes in climate and human usage and to develop new varieties adapted to biotic and abiotic stresses. Therefore, for the improvement of the long‐term breeding program, potato breeders should choose a proper weight on He accounting to their parameters for a subsequent long‐term improvement in genetic gain and nevertheless adaptability of the breeding program. In detail, to reach high long‐term genetic gain while simultaneously maintaining a certain diversity, ${\mathrm{EUCD}}_{\left(1,50|500\right)}$ can be used for cases with no, mild, and moderate dominance effects, where ${\mathrm{EUCD}}_{\left(1,2500\right)}$ seems to be appropriate for cases with strong dominance effects. However, ${\mathrm{EUCD}}_{\left(1,2500\right)}$ or ${\mathrm{EUCD}}_{\left(1,5000\right)}$ can be utilized if the main breeding goals are to keep maximum diversity and to reach a certain genetic gain for the cases with moderate or strong dominance effects. Therefore, the choice of the most appropriate weight on diversity in EUCD depends not only on the genetic architecture of $T_{t}$, but also on the breeder's objectives.

### 4.5 Assumptions of the present study

In this study, we assume that the parental haplotype phase is known, and, therefore, the progeny variance can be predicted by in silico progenies (Bernardo, 2014; Mohammadi et al., 2015; Miller et al., 2023). However, also with current methodology (e.g., Sun et al. 2022), the assessment of the haplotype phase is costly. Thus, in current breeding programs, the possibility of estimating the progeny mean is based on mid‐parent performance. In this study, MPV had a higher accuracy in predicting progeny mean compared to MEGV‐P or MEBV‐P because the heritability (0.73) is higher than the PA (0.5). However, if heritability is lower than PA, the advantage of MPV compared to MEBV‐P and MEGV‐P disappears. For example, the heritability at early breeding stages is lower than the one at late breeding stages, because the former has less experimental locations and replications than the latter. Therefore, if the candidate parents are selected from early breeding stages, the superiority of MPV over MEBV‐P or MEGV‐P will diminish.

Wolfe et al. (2021) and Werner et al. (2023) predicted the progeny mean with the formula based on allele frequencies of parents and considering additive and dominance effects from Falconer and Mackay (1996) in heterozygous diploid crops. In empirical data, Wolfe (2021) found no improvement in progeny mean prediction accuracy when using MEGV estimated by the formula, when comparing to MEBV estimated from mid‐parental values. In contrast, Werner et al. (2023) indicated that genetic gain was improved using MEGV estimated by the formula for cross selection. This improvement was particularly evident for traits with dominance effects. Therefore, one possibility to improve the prediction of progeny mean in future research entails developing the formula to estimate progeny mean and variance in autotetraploid species. Furthermore, ${\mathrm{He}}_{\mathrm{per}-\mathrm{cross}}$ based on simulated progenies is highly correlated with ${\mathrm{He}}_{\mathrm{per}-\mathrm{cross}}$ based on parental genotypic information (data not shown). Thus, the lack of information about haplotype phase does not affect the ability to quantify genome‐wide diversity of a cross.

An alternative method to consider genome‐wide diversity while selecting new crosses for the next breeding cycle was developed by Gorjanc et al. (2018) and Allier et al. (2019). Their approach is called optimal cross‐selection (OCS). The OCS relies on an optimization algorithm to select a group of biparental crosses that maximize the cross performance with a certain constraint of genetic diversity on the selected population of individuals who serve as parents. To search for an optimal group of crosses, this method requires an optimization process by evolutionary algorithms (e.g., Storn & Price, 1997; Whitley, 1994), which were inspired by natural selection. Appropriate parameters must be set up in these algorithms to avoid reaching a solely local optimal solution. These parameters include inter alia population size, crossover, mutation, selection, and the number of iterations to terminate the optimization process. Obviously, this optimization process leads to very intensive computation compared to our proposed EUCD methods. EUCD only requires ranking the performance considering the designed genome‐wide diversity among all possible crosses to reach high genetic gain while maintaining diversity. The intensive demand of computation of OCS is even more pronounced with an increasing number of markers, repetitions, and candidates. Our study considered between 2 and 24 times more SNP and triple repetition numbers compared to the studies conducted by Gorjanc et al. (2018) and Allier et al. (2019). In addition, the number of all possible solutions for selecting 300 from 1790 possible crosses in this study is infinite, and far greater than the ones in previous studies. Thus, OCS has not been assessed in this study. However, the comparison of performance between the two methods requires further research.

### 4.6 Summary

The present study demonstrated that implementing GS with optimal selection intensity per stage enhances both short‐ and long‐term gain from selection compared to a typical tetraploid potato breeding program based solely on PS. In addition, for autotetraploid and heterozygous crops, the prediction of progeny mean considering not only additive but also dominance effects (MEGV‐O) is advantageous. This approach results in the highest prediction accuracy to predict progeny mean and has the highest genetic gain among all mean‐based CS methods. Furthermore, combining UC and genome‐wide diversity (EUCD) by a linear combination achieved the same level of long‐term genetic gain in a tetraploid potato breeding program. However, it simultaneously preserved higher diversity, a certain degree of genetic variance, and a lower number of fixed QTLs compared to MEGV‐O and UC. In our results, although EUCD with a low weight can reach the highest genetic gain, different genetic architectures of $T_{t}$ and the breeder's objectives require choosing different weights on genome‐wide diversity to achieve a high genetic gain and simultaneously preserve sufficient diversity. These results can provide breeders with a concrete method to improve their potato breeding programs.

## AUTHOR CONTRIBUTIONS

**Po‐Ya Wu**: Conceptualization; data curation; formal analysis; writing—original draft. **Benjamin Stich**: Conceptualization; funding acquisition; project administration; supervision; review and editing. **Stefanie Hartje**: Funding acquisition; resources; review and editing. **Katja Muders**: Funding acquisition; resources; review and editing. **Vanessa Prigge**: Funding acquisition; resources; review and editing. **Delphine Van Inghelandt**: Conceptualization; funding acquisition; project administration; supervision; writing—original draft; review and editing.

## CONFLICT OF INTEREST STATEMENT

The authors declare no conflicts of interest.

## Supporting information

Table S1: Dimensioning of a standard potato breeding program that exclusively relies on phenotypic selection.
Table S2: The mean and standard deviation (sd) of the genetic gain and the genetic variance for the target trait, as well as genome‐wide diversity measured by expected heterozygosity (He) at cycle 30 across 30 simulation runs. Simulations were based on the Optimal‐GS selection strategy for different cross‐selection methods (MPV, MEGV‐O, EUC, and EUCD), and different genetic architectures of the target traits (no, mild, moderate, and strong dominance effects). The details of EUC and EUCD are shown in Table 1.
Figure S1: Graphical illustration of the three selection strategies: (1) Standard‐PS, where PS is phenotypic selection, (2) Optimal‐PS, and (3) Optimal‐GS, where genomic selection (GS) is applied at SH and A stages (GS‐SH:A) with the prediction accuracy of the GS model of 0.5 and the correlation between the two traits of 0.15. $p_{1}$ to $p_{5}$ are the selected proportions from SL to SH, SH to A, A to B, B to C, and C to D, respectively, where SL, SH, A, B, C, and D represent the stages of seedling, single hills, A, B, C, and D clones. The selected proportions for the strategy (1) Standard‐PS follow the standard potato breeding program (Wu et al., 2023). The optimal selected proportions for (2) Optimal‐PS and (3) Optimal‐GS are determined by achieving the maximum short‐term genetic gain. $\alpha_{k}$ and $N_{1}$ are the weight of genomic selection relative to phenotypic selection and the number of clones at seedling stage, respectively. The details of implementing GS in a breeding program are shown in Figure S2.
Figure S2: Graphical illustration of the standard as well as the six selection strategies that include genomic selection that were examined in our study. $p_{1}$ to $p_{5}$ are the selected proportions from SL to SH, SH to A, A to B, B to C, C to D, respectively, where SL, SH, A, B, C, and D represent the stages of seedling, single hills, A, B, C, and D clones. $\alpha_{k}$ is the proportion of clones selected by PS to be genotyped in stage k and $N_{k}$ is the number of clones of the respective stage.
Figure S3: The comparison of maximum genetic gain (dashed red line, see discussion for details) and the genetic gain of different mean‐based cross‐selection methods across different genetic architectures of the target trait (no, mild, moderate, and strong dominance effects).

## Data Availability

The datasets generated or analyzed during this study and R scripts are available from the authors upon request.
