# Supplementary material for: Optimal implementation of genomic selection in clone breeding programs exemplified in potato: II. Effect of selection strategy and cross‐selection method on long‐term genetic gain
Source: Plant Genome. 2025 Feb 18;18(1):e70000. doi: 10.1002/tpg2.70000 (PMC11835509; doi:10.1002/tpg2.70000)
Supplement: Supplementary file 1 [file TPG2-18-e70000-s001.pdf]

SUPPLEMENTARY MATERIAL

Method S1: Simulation of true genetic values for $\mathrm{T_a}$ (Wu et al., 2023)

The true genetic values for $\mathrm{T_a}$ were generated by $\mathrm{TGV_{T_a}} = \mathrm{TGV_{T_t}} + \epsilon_\mathrm{r}$, where ϵ_r was the residual value following a normal distribution $N(0, \sigma^2_{\epsilon_\mathrm{r}})$, with

$$\sigma^2_{\epsilon_\mathrm{r}} = \frac{1}{n-2}\frac{1-\mathrm{r}^2}{\mathrm{r}^2}\sum_{i=1}^{n}(\mathrm{TGV_{T_t(i)}} - \overline{\mathrm{TGV}}_\mathrm{T_t})^2 \quad [1]$$

determined by the degree of the correlation r between $\mathrm{T_a}$ and $\mathrm{T_t}$, where n was the number of clones at seedling stage, $\mathrm{TGV_{T_t(i)}}$ the TGV for $\mathrm{T_t}$ of the ith clone, and $\overline{\mathrm{TGV}}_\mathrm{T_t}$ the average of $\mathrm{TGV_{T_t}}$ at seedling stage.

Method S2: Optimal selected proportions (Wu et al., 2023)

To determine the optimal selected proportions maximizing a short-term genetic gain under a given budget, a general linear cost function to aggregate all costs across all stages in the breeding program was created:

$$\begin{aligned}\mathrm{Budget} &= \sum_{j=1}^{6} \mathrm{N}_j \times \mathrm{cost_{pheno(\mathit{j})}} \times \mathrm{L}_j + \mathrm{N_{GS}} \times \mathrm{cost_{geno}} \\ &= \sum_{j=1}^{5} \frac{\mathrm{N}_6}{\Pi_{k=j}^{5}\mathrm{p}_k}\mathrm{cost_{pheno(\mathit{j})}}\mathrm{L}_j + \mathrm{N}_6\mathrm{cost_{pheno(6)}}\mathrm{L}_6 + \frac{\mathrm{N}_6\mathrm{cost_{geno}}\alpha_m}{\Pi_{k=m}^{5}\mathrm{p}_k},\end{aligned} \quad [2]$$

where N_j was the number of clones at stage j, $\mathrm{cost_{pheno(\mathit{j})}}$ the cost for phenotypic evaluation at stage j, $\mathrm{N_{GS}}$ the number of genotyped clones, L_j the number of locations at stage j, and $\mathrm{cost_{geno}}$ the genotyping cost. In addition, p_k was the selected proportion from stage $j(m)$ to stage $j(m)+1$, where m was the stage in which GS was applied first. α_k was the the weight of genomic selection relative to phenotypic selection. For more details, $m = 1$ referred to GS-SL, GS-SL:SH and GS-SL:SH:A; $m = 2$

for GS-SH and GS-SH:A; and $m = 3$ for GS-A, where (1) GS-SL, (2) GS-SL:SH, (3) GS-SL:SH:A, (4) GS-SH, (5) GS-SH:A, and (6) GS-A were that GS is applied at (1) seedling, (2) seedling and single hills, (3) seedling, single hills, and A clone, (4) single hills, (5) single hills and A clone, and (6) A clone stage(s), respectively (Figure S2). The GS strategies with optimal selected proportions will be named Optimal-GS hereafter.

The optimal selected proportions was determined by a grid search across the permissible space of p_2 to p_5 and α_k for a set of given input parameters. The latter included the number of tested clones at D clone stage (N_6), the GS strategy, the phenotyping and genotyping costs, L, r, variance components of T_t, $H^2_{T_a}$, and the total budget. In this study, N_6 was set to 60. In the grid search, any p_k varied between 0.1 and 0.5 in increments of 0.05 to avoid too strong/weak selections. α_k varied from 0.4 to 0.9 in increments of 0.1 for the strategies GS-SL, GS-SL:SH, and GS-SL:SH:A; and from 0.2 to 0.9 in increments of 0.1 for the other strategies. Consequently, in each permissible allocation, p_1 was completely determined by equation [2] under the constrained budget and the given input parameters. Subsequently, the mean genetic gain across 1,000 simulation runs in one breeding cycle was calculated for each permissible allocation of the grid search. The optimal selected proportions were determined when achieving the maximum short-term genetic gain across all permissible allocations of the grid search.

Reference

Wu, P.-Y., Stich, B., Renner, J., Muders, K., Prigge, V., and van Inghelandt, D. (2023). Optimal implementation of genomic selection in clone breeding programs—Exemplified in potato: I. Effect of selection strategy, implementation stage,

and selection intensity on short-term genetic gain. *The Plant Genome*, page e20327.

Table S1: Dimensioning of a standard potato breeding program that exclusively relies on phenotypic selection.

Stage	Number of clones	Number of locations	Phenotyping cost per clone and plot (€)	Cost per stage (€)
Seedling	300,000 (N_1)	1	1.4	420,000
Single hills	100,000 (N_2)	1	1.4	140,000
A clone	10,000 (N_3)	1	1.4	14,000
B clone	1,500 (N_4)	2	25	75,000
C clone	300 (N_5)	3	25	22,500
D clone	60 (N_6)	4	25	6,000
Sum				677,500

Table S2: The mean and standard deviation (sd) of the genetic gain and the genetic variance for the target trait, as well as genome-wide diversity measured by expected heterozygosity (He) at cycle 30 across 30 simulation runs. Simulations were based on the Optimal-GS selection strategy for different cross-selection methods (MPV, MEGV-O, EUC and EUCD), and different genetic architectures of the target trait (no, mild, moderate, and strong dominance effects). The details of EUC and EUCD are shown in Table 1.

Case	Scale	Cross-selection method	Genetic gain	Genetic variance	He
			mean | sd | rank[1] | group[2]	mean | sd | rank[1]	mean | sd | rank[1]
No dominance	-	Optimal-GS: MPV	674.86 | 45.38 | 6 | b	25.54 | 7.17 | 6	0.1354 | 0.0236 | 9
		MEGV-O	682.91 | 36.83 | 4 | ab	20.55 | 7.12 | 9	0.1419 | 0.0211 | 8
	A	$EUC_{(1,0)}$=UC	690.42 | 34.25 | 2 | ab	20.50 | 6.79 | 10	0.1438 | 0.0241 | 7
		$EUCD_{(1,50)}$	688.26 | 37.24 | 3 | ab	22.27 | 5.59 | 8	0.1462 | 0.0241 | 6
	B	$EUC_{(10,0)}$	682.03 | 35.41 | 5 | ab	27.62 | 7.19 | 5	0.1307 | 0.0216 | 10
		$EUCD_{(1,500)}$	697.52 | 35.45 | 1 | a	25.41 | 5.29 | 7	0.1762 | 0.0193 | 5
	C	$EUC_{(50,0)}$	510.83 | 41.30 | 7 | c	158.38 | 37.96 | 4	0.2125 | 0.0255 | 4
		$EUCD_{(1,2500)}$	474.79 | 32.86 | 9 | d	201.18 | 59.78 | 2	0.4291 | 0.0106 | 2
	D	$EUC_{(100,0)}$	479.07 | 31.80 | 8 | d	195.13 | 45.51 | 3	0.2432 | 0.0287 | 3
		$EUCD_{(1,5000)}$	345.34 | 29.90 | 10 | e	277.26 | 152.84 | 1	0.4962 | 0.0107 | 1
Mild dominance	-	Optimal-GS: MPV	589.13 | 24.70 | 6 | b	165.21 | 29.62 | 6	0.3686 | 0.0146 | 10
		MEGV-O	630.46 | 21.73 | 3 | a	155.91 | 32.38 | 8	0.3871 | 0.0120 | 8
	A	$EUC_{(1,0)}$=UC	634.86 | 20.86 | 2 | a	152.32 | 29.24 | 10	0.3880 | 0.0132 | 7
		$EUCD_{(1,50)}$	635.53 | 18.70 | 1 | a	154.53 | 28.70 | 9	0.3896 | 0.0107 | 6
	B	$EUC_{(10,0)}$	607.07 | 24.09 | 5 | b	187.34 | 32.32 | 5	0.3833 | 0.0130 | 9
		$EUCD_{(1,500)}$	627.08 | 18.74 | 4 | a	161.12 | 36.10 | 7	0.3963 | 0.0103 | 4
	C	$EUC_{(50,0)}$	538.28 | 19.13 | 8 | c	233.10 | 46.12 | 4	0.3942 | 0.0122 | 5
		$EUCD_{(1,2500)}$	539.83 | 21.64 | 7 | c	316.13 | 86.45 | 2	0.4611 | 0.0075 | 2
	D	$EUC_{(100,0)}$	517.75 | 26.58 | 9 | d	245.84 | 39.13 | 3	0.4026 | 0.0147 | 3
		$EUCD_{(1,5000)}$	437.93 | 21.82 | 10 | e	375.27 | 101.12 | 1	0.5045 | 0.0096 | 1

Case	Scale	Cross-selection method	Genetic gain	Genetic variance	He
			mean | sd | rank[1] | group[2]	mean | sd | rank[1]	mean | sd | rank[1]
Moderate dominance	-	Optimal-GS: MPV	615.59 | 29.91 | 8 | cd	536.13 | 110.22 | 6	0.4273 | 0.0102 | 10
		MEGV-O	722.54 | 25.61 | 3 | a	483.54 | 97.25 | 9	0.4442 | 0.0087 | 5
	A	$EUC_{(1,0)}$=UC	731.97 | 24.21 | 1 | a	473.95 | 96.69 | 10	0.4400 | 0.0072 | 7
		$EUCD_{(1,50)}$	722.47 | 26.83 | 4 | a	508.45 | 98.52 | 8	0.4446 | 0.0088 | 4
	B	$EUC_{(10,0)}$	683.29 | 21.74 | 6 | b	616.05 | 115.14 | 5	0.4346 | 0.0096 | 9
		$EUCD_{(1,500)}$	731.00 | 25.33 | 2 | a	510.10 | 108.94 | 7	0.4504 | 0.0086 | 3
	C	$EUC_{(50,0)}$	617.29 | 29.37 | 7 | c	633.84 | 104.73 | 4	0.4364 | 0.0110 | 8
		$EUCD_{(1,2500)}$	693.48 | 28.76 | 5 | b	635.23 | 120.92 | 3	0.4788 | 0.0054 | 2
	D	$EUC_{(100,0)}$	603.47 | 27.35 | 10 | d	685.91 | 188.11 | 2	0.4402 | 0.0097 | 6
		$EUCD_{(1,5000)}$	611.82 | 27.36 | 9 | cd	799.24 | 118.21 | 1	0.5038 | 0.0060 | 1
Strong dominance	-	Optimal-GS: MPV	709.57 | 29.15 | 10 | d	1150.66 | 223.20 | 5	0.4459 | 0.0089 | 10
		MEGV-O	863.63 | 37.04 | 4 | a	937.20 | 192.78 | 10	0.4598 | 0.0082 | 6
	A	$EUC_{(1,0)}$=UC	866.76 | 34.68 | 2 | a	1012.18 | 189.86 | 8	0.4601 | 0.0074 | 5
		$EUCD_{(1,50)}$	866.11 | 25.14 | 3 | a	1035.21 | 240.35 | 7	0.4616 | 0.0092 | 4
	B	$EUC_{(10,0)}$	797.36 | 33.52 | 6 | b	1157.18 | 237.24 | 4	0.4504 | 0.0065 | 7
		$EUCD_{(1,500)}$	873.48 | 28.85 | 1 | a	978.41 | 199.76 | 9	0.4654 | 0.0067 | 3
	C	$EUC_{(50,0)}$	733.52 | 41.32 | 8 | c	1385.26 | 240.45 | 2	0.4479 | 0.0085 | 9
		$EUCD_{(1,2500)}$	859.17 | 36.96 | 5 | a	1071.70 | 230.82 | 6	0.4840 | 0.0054 | 2
	D	$EUC_{(100,0)}$	720.28 | 36.26 | 9 | cd	1352.71 | 289.42 | 3	0.4484 | 0.0107 | 8
		$EUCD_{(1,5000)}$	796.02 | 25.44 | 7 | b	1456.23 | 291.13 | 1	0.5037 | 0.0047 | 1

[1] The number after the sd represent the rank across these cross-selection methods within a specific genetic architecture.

[2] The letters after the rank represent the significance groups ($P < 0.05$) across these cross-selection methods within a specific genetic architecture.

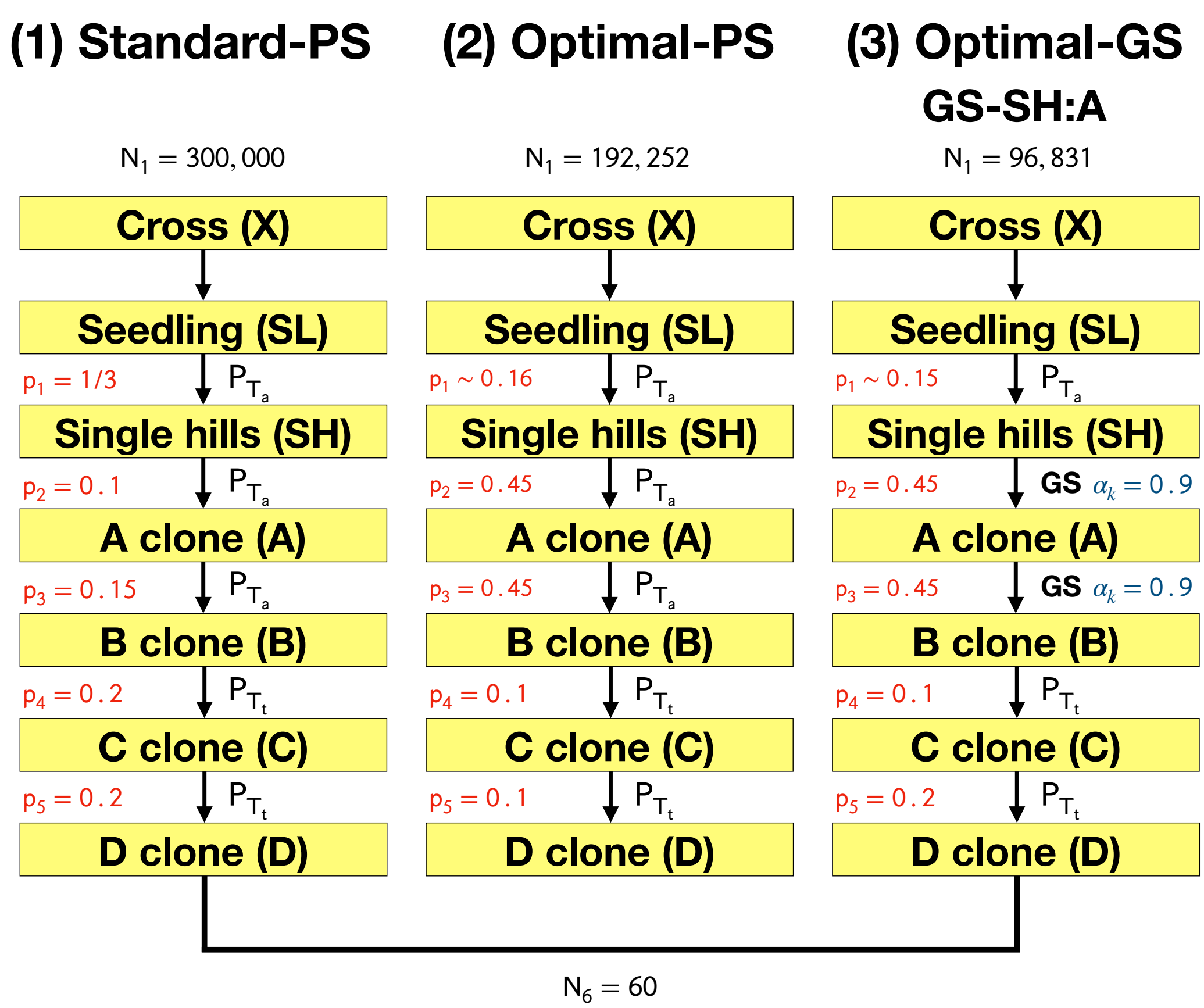

Figure S1: Graphical illustration of the three selection strategies: (1) Standard-PS, where PS is phenotypic selection, (2) Optimal-PS, and (3) Optimal-GS, where genomic selection (GS) is applied at SH and A stages (GS-SH:A) with the prediction accuracy of the GS model of 0.5 and the correlation between the two traits of 0.15. p_1 to p_5 are the selected proportions from SL to SH, SH to A, A to B, B to C, and C to D, respectively, where SL, SH, A, B, C, and D represent the stages of seedling, single hills, A, B, C, and D clones. The selected proportions for the strategy (1) Standard-PS follow the standard potato breeding program (Wu et al., 2023). The optimal selected proportions for (2) Optimal-PS and (3) Optimal-GS are determined by achieving the maximum short-term genetic gain. α_k and N_1 are the weight of genomic selection relative to phenotypic selection and the number of clones at seedling stages, respectively. The details of implementing GS in a breeding program are shown in Figure S2.

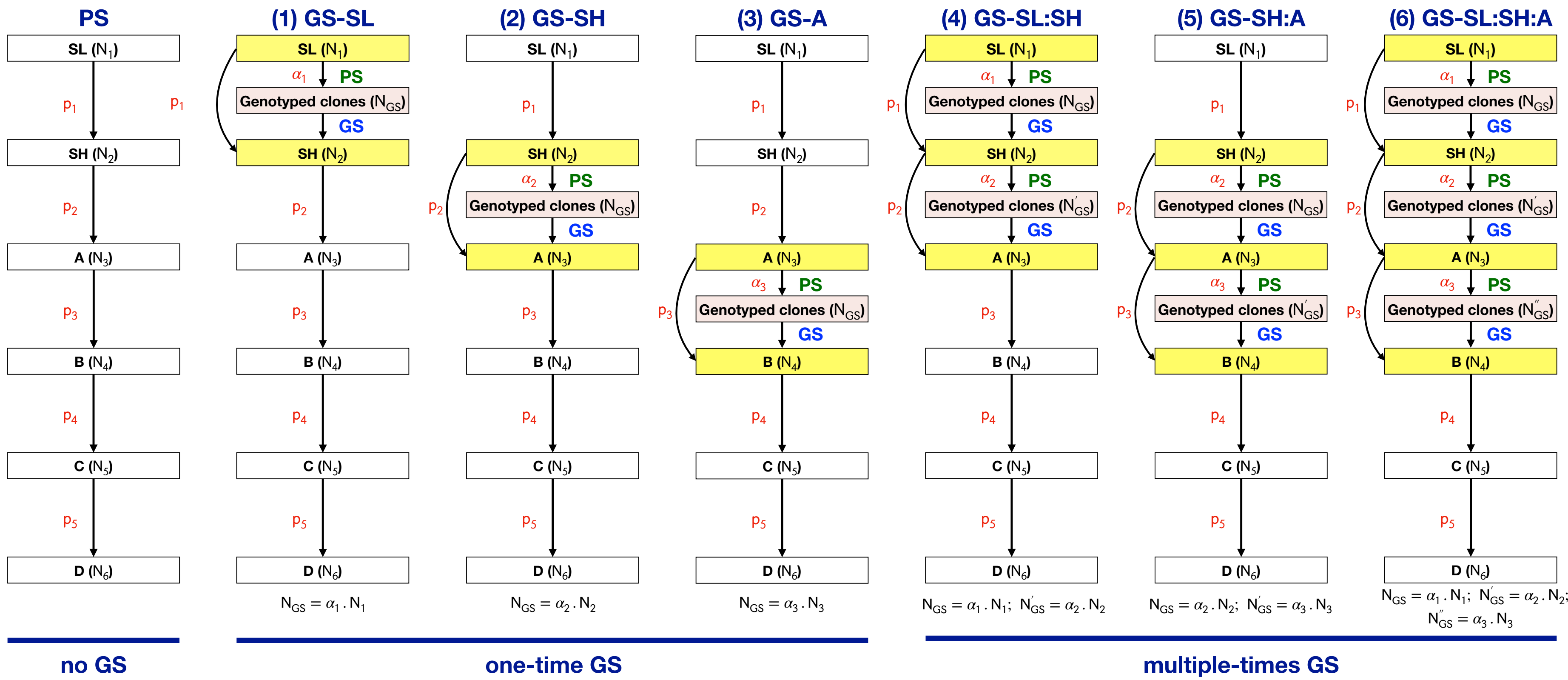

Figure S2: Graphical illustration of the standard as well as the six selection strategies that include genomic selection that were examined in our study. p_1-p_5 are the selected proportions from SL to SH, SH to A, A to B, B to C, and C to D, respectively, where SL, SH, A, B, C, and D represent the stages of seedling, single hills, A, B, C, and D clones. α_k the proportion of clones selected by PS to be genotyped in stage k and N_k is the number of clones of the respective stage.

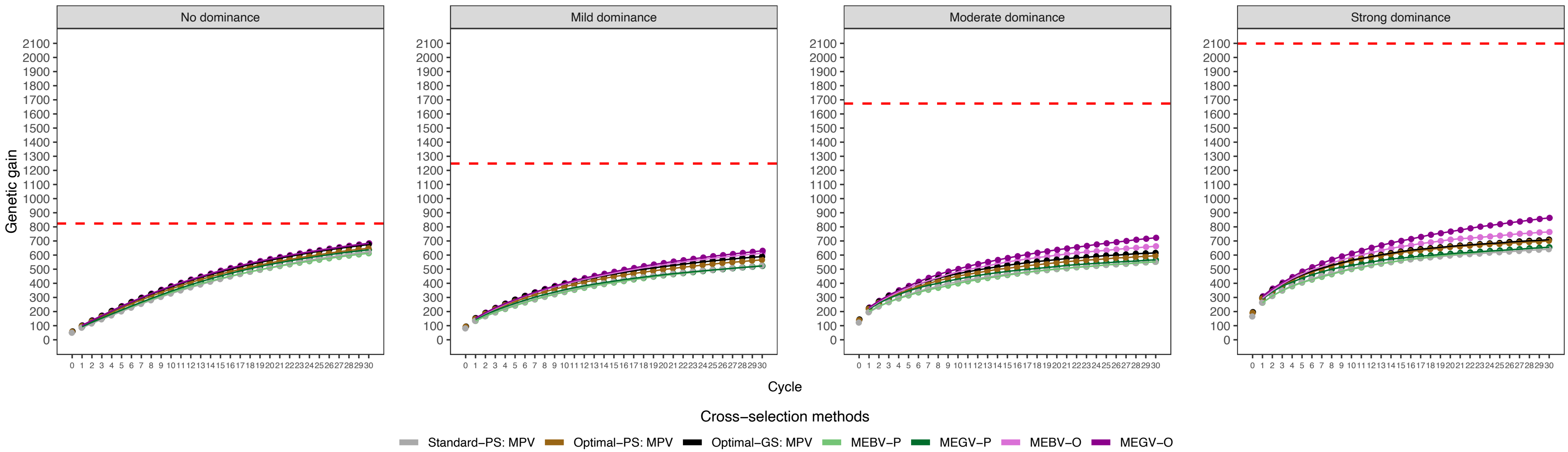

Figure S3: The comparison of maximum genetic gain (dashed red line, see discussion for details) and the genetic gain of different mean-based cross-selection methods across different genetic architectures of the target trait (no, mild, moderate, and strong dominance effects).
